# Leveraging genetics to optimize rehabilitation outcomes after spinal cord injury: contemporary challenges and future opportunities

**DOI:** 10.3389/fgene.2024.1350422

**Published:** 2024-02-13

**Authors:** Andrew Park, Ryan Solinsky

**Affiliations:** ^1^ Craig Rehabilitation Hospital, Englewood, CO, United States; ^2^ Mayo Clinic, Rochester, MN, United States

**Keywords:** spinal cord injury, genetics, paralysis, outcomes, rehabilitation

## Introduction

Genetic analyses have revolutionized multiple fields of medicine, fulfilling many of the promises of targeted treatments with improved patient survival and disease-free progression. Wanting to see similar personalization, rehabilitation medicine has further waded into these waters, with early efforts beginning to bear fruit.

Recovery from stroke has been tied to an intricate interaction of age, severity, and genetics, with BDNF Val66met polymorphisms playing a prominent role. Balkaya and Cho (2019) Other candidate genes have been identified through genome-wide association studies (GWAS), (Kessler and Schunkert, 2019; Mola-Caminal et al., 2019) opening up not only improved prognostication, but potential for more targeted therapies. Similarly in traumatic brain injury, GWAS have identified key genetic loci which seem to play a role in recovery trajectories. Cortes and Pera (2021); Kals et al. (2022) Even conditions such as osteoarthritis have identified heritability patterns using large genetic databases to determine individuals at increased risk Aubourg et al. (2022).

However, within spinal cord injury (SCI) medicine, there has yet to be a pivotal study which changes clinical care. Herein, we discuss the challenges genetic analyses have in SCI medicine and offer directions for ways forward.

## Contemporary challenges

Not unique to many rare conditions, SCI has a dilemma with heterogeneity and numbers. In the United States, there are approximately 17,500 acute traumatic SCIs per year, with only half of those being admitted to a specialized inpatient rehabilitation facility where collection of comprehensive outcomes necessary for genetic-based studies typically will be captured. National Spinal Cord Injury Statistical Center (2017) This relatively small number is often further subdivided into motor/sensory completeness and varied neurological levels of injury. Couris et al. (2010) To add further, many standardized outcomes such as classification with the International Standards exam, or clinically relevant endpoints (admission venous thromboemboli screening, spasticity, neuropathic pain quantification) are not uniformly employed. Even the most widely used endpoint, the International Standards for Neurological Classifications of Spinal Cord Injury (ISNCSCI) exam, (Rupp et al., 2021) requires specialized training and is subject to reliability and validity error dependent on the examiner’s skill and injury characteristics. Hales et al. (2015) These measures with some objectivity differ from laboratory endpoints such as A1C or incidence of clearly defined clinical complications such as myocardial infarction—which lend themselves far better to genetic studies.

Taken together, the lack of significant progress in clinical SCI genetics studies can be traced to lack of adequate genetic data and lack of uniform endpoints of interest. Further, once a patient with SCI discharges from initial inpatient rehabilitation, timing of follow up and outcome recordings are often non-standardized. Individuals with SCI also commonly have heavy burdens of secondary medical complications related to paralysis from increased risk of infections such as urinary tract infections and pneumonias, to musculoskeletal conditions that impede effective scoring of motor and sensory neurorecovery such as spasticity, (Sangari and Perez, 2022), muscle contractures, (Diong et al., 2012) and fragility fractures. Bethel et al. (2016) Given their frequency (Cardenas and Hooton, 1995) and long-term impacts on recovery, (Jaja et al., 2019) these additional confounding factors may skew outcomes analyzed in studies of fixed genetic data.

Many of the challenges of compounding secondary complications which confound the outcome of interest can be addressed with sufficient sample size, across a large regional area of interest (i.e., national or continental) to demonstrate the independent effect of the genetic data. Thus, the dilemma continues with understanding when to group a relatively rare condition to have sufficient sample size, and when/where to stratify to control for heterogeneity.

## Future opportunities

However, not all is bleak. As previously stated, these challenges are not unique to SCI research and modelling successful efforts in other rare diseases demonstrates several solutions. A daunting but necessary effort is the pooling of resources and deep collaborations among clinical and research sites who provide care and research to individuals with SCI. The best example of these efforts to date include several national and international patient registries including the United States SCI Model Systems, the Canadian National SCI Registry, European Multicenter Study about SCI, Australian SCI Registry, and many other national registries. The coordinated efforts of the epidemiological data collected by these registries have translated to the improved understanding of needs of persons with SCI which have led to practice change and research focus. Germaine to genetics research after SCI, these collaborative efforts must expand to the meaningful collection, storage, and distribution of biosamples. Examples of such biobanking or biorepository efforts are noted across multiple rare diseases such as the International Rare Diseases Research Consortium (IRDiRC). Cutillo et al. (2017) Multiple smaller entities in SCI are further focused on this problem, setting up local and hospital system-wide biobanks of well characterized individuals with SCI and standardized outcome measures. To date, these independent biobanks commonly have in the low hundreds of samples, opening potential avenues for pooling data through open data repositories, (Torres-Espín et al., 2022) use of common data elements, (Biering-Sørensen et al., 2015) or collaborative agreements.

Additionally, while large population-based studies will likely continue to be challenging, differing study designs may be helpful. Repeated measures within the same individual to identify acquired mutations is a potentially fruitful approach to better understand the influence of SCI. Adaptive trial designs further allow for real time assessment of various confounding factors into an outcome of interest. Mulcahey et al. (2020).

A critical area of improvement in SCI research to facilitate such alternative study designs is identifying/validating surrogate biomarkers. Collaborative, prospective endeavors such as the SCI-TRACK program have begun these efforts, validating the use of GFAP and Neurofilament-A-Light Chain as the first two biomarkers of neurorecovery under the strict FDA definition of a qualified prognostic biomarker. Singular other more stringent biomarkers, such as the use of neurophysiological measurements of motor recovery in recent clinical trials (NCT05965700), would also provide more nuanced data with which to identify genetic associations. Although biomarkers are never a replacement for true clinical outcomes, they reduce uncertainty and provide anchor points to assist researchers to interpret complex interactions, as well as practically accelerate when and how a biosample may be utilized. Addressing these challenges by potentially pursuing some of these future opportunities will be important to advance genetics studies in SCI medicine. Fortunately, the path to population level genetics research is well established in other conditions. The challenge for the SCI community is developing the infrastructure and collaborations to bring it to fruition.
